# 

**Published:** 1899-12

**Authors:** 


### NOTICES.

### Toledo Dental Society.

The regular meetings of the Toledo Dental Society will be held Friday afternoons and evenings of the following dates: October 13th, November 10th, December 8th, January 12th, February 9th, March 9th, April 13th, May 11th, June 8th.

L. T. CANFIELD, *Secretary.*

### EDITORIAL.

### The National School of Dental Technics.

The next meeting of this organization will be held at the Continental Hotel in Philadelphia at 10 o'clock, A. M., Wednesday, December 27th, and continued three days. Every teacher in the dental schools should be present at that convocation. A very good program will be presented. It is as follows:

WEDNESDAY, 27TH.

10 A.M.—Preliminary Organization—Payment of Dues, Report of Executive Officers, Executive Business.

10:30 A.M.—Report of Committees on Syllabi; Operative Technic, Dr. T. E. Weeks; Prosthetic Technic, Dr. H. J. Goslee.

11:30 A.M.—President's Address, Dr. N. S. Hoff; Discussion, Drs. E. C. Kirk and H. A. Smith.

2 P.M.—Syllabus for Dental Curriculum, Dr. F. D. Weisse; Discussion, Drs. S. H. Guilford, M. W. Foster and G. V. I. Brown.

3:30 P.M.—Dental Pedagogics, Dr. A. E. Webster; Discussion, Drs. C. N. Johnson, R. H. Hofheinz and L. G. Noel.

7:30 P.M.—Address—Manual Training, Prof. J. Liberty Tadd.

8:30 P.M.—The Use of the Lantern in Teaching, Dr. M. H. Cryer.

THURSDAY, 28TH.

10 A.M.—Orthodontia Technic, Dr. C. S. Case; Discussion, Drs. C. D. Lukins, Grant Molyneaux and B. Holly Smith.

11:30 A.M—Root Preparation Technic, Dr. H. J. Goslee; Discussion, Drs. I. N. Broomell, H. W. Morgan and H. P. Carlton.

2 P.M.—The Use of the Blackboard in Technic, Dr. A. D. Gritman; Discussion, Drs. C. J. Essig and H. W. Arthur.

3:30 P.M.—Operatory Methods, Dr. W. H. Whitslar; Discussion, Drs. W. E. Wilmont and D. M. Cattell

5 P.M—Election of Officers.

FRIDAY, 29TH.

10 A.M.—The Use of Text-Books in Class Work, Dr. Otto Arnold; Discussion, Drs. G. E. Hunt and H. B. Tileston;

11 A.M.—Symposium of fifteen-minute papers on our Text-Books: Operative Dentistry, Dr. G. V. Black; Prosthetic Dentistry, Dr. I. N. Broomell; Dental Pathology, Dr. A. H. Thompson; Dental Therapeutics, Dr. James Truman; Oral Surgery, Dr. T. W. Brophy.

1 P.M.—Adjournment.

This is an array of talent that certainly ought to have strong attraction for those who are engaged in teaching in dental schools. None whom circumstances do not compell to remain away can afford to be absent on that occasion. Let every one who can, be present and prepared to contribute to the interest and benefit of the occasion.

Undoubtedly all who are receiving professional education in our schools will be greatly benefited by what will be accomplished at this meeting. Let arrangements everywhere be made with reference to attendance upon this meeting.

### CONTENTS.

### COMMUNICATIONS.

NOTICES.

Toledo Dental Society ............................................... 579

EDITORIAL.

The National School of Dental Technics .............................. 579

### THE DENTAL REGISTER,

A MONTHLY MAGAZINE DEVOTED TO THE INTERESTS OF THE

### DENTAL PROFESSION.

*Terms Reduced to* $2.00 *Per Year. Single Copies,* 25 *Cents.*

### EDITED BY PROF. J. TAFT, D.D.S.

—AND—

### Published by SAM'L A. CROCKER & CO., Ohio Dental and Surgical Depot.

The volume commences in January and closes in December.

The REGISTER being issued monthly is always fresh, therefore Indispensable to the practitioner who desires to keep posted up with the new inventions and improvements which are daily developed. Neither expense nor effort will be spared to give value and variety to its contents. Articles will be illustrated with well executed engravings whenever they will add to the interest or assist to explanation.

Its extensive circulation and frequency of issue make it one of the BEST DENTAL ADVERTISING MEDIUMS in the United States.

All subscriptions must begin with January or July.

Local or special notices, five lines or less, will be inserted for $3 each insertion; over five lines, 50 cts. per line.

All advertising bills payable in advance, or at furthest, after the issue of the first number containing the advertisement. All transient advertisements to be paid for in advance.

☞CONTRIBUTIONS SOLICITED.

Exclusively Editorial Communications should be addressed to the Editor.

The latest number of the REGISTER may be ordered with or without other goods at any time, and all letters should be addres[illegible]

